# Sarcopenic obesity does not impair lower limb strength and physical performance in sufficiently active older adults: a cross-sectional study

**DOI:** 10.1038/s41598-024-53538-7

**Published:** 2024-02-06

**Authors:** Leonardo Santos Lopes da Silva, Pedro Pugliesi Abdalla, Pablo Jorge Marcos-Pardo, Vicente Romo‑Perez, Jose Luis Garcia‑Soidan, Jorge Mota, Dalmo Roberto Lopes Machado

**Affiliations:** 1 School of Physical Education and Sport of Ribeirao Preto, University of São Paulo, Bandeirantes Avenue No 3900, University Campus - Monte Alegre, Ribeirao Preto, SP 14030-680 Brazil; 2 Study and Research Group in Anthropometry, Training, and Sport (GEPEATE), University of São Paulo, School of Physical Education and Sport of Ribeirão Preto, Ribeirão Preto, Brazil; 3 Department of Education, Faculty of Educational Sciences, University of Almería, 04120 Almería, Spain; 4 CERNEP Research Center, SPORT Research Group (CTS-1024), University of Almería, 04120 Almería, Spain; 5 Active Aging, Exercise and Health/HEALTHY-AGE Network, Consejo Superior de Deportes (CSD), Ministry of Culture and Sport of Spain, 28040 Madrid, Spain; 6 Faculty of Education and Sport Sciences, University of Vigo, Vigo, Spain; 7 The Research Centre in Physical Activity, Health, and Leisure (CIAFEL), University of Porto, Porto, Portugal; 8 Laboratory for Integrative and Translational Research in Population Health (ITR), University of Porto, Porto, Portugal; 9 Ribeirão Preto College of Nursing, University of São Paulo, Ribeirão Preto, SP Brazil; 10 ESEC - Universidade do Algarve, Campus da Penha, Faro, Portugal

**Keywords:** Geriatrics, Risk factors

## Abstract

This study investigated the associations of sarcopenic obesity (SO) with muscle strength and physical performance in sufficiently active older adults. Data from 72 older sarcopenic obese adults classified as sufficiently active were analyzed. Participants were categorized into four groups based on sex and SO status. Muscle strength/physical performance tests were compared using independent sample t-tests. Multiple linear regression and binary logistic regression were performed to examine the associations between SO and muscle strength and physical performance, adjusting for confounding variables. Only handgrip strength showed differences between SO groups, regardless of sex (*p* < 0.05). SO negatively explained the variability of handgrip strength (*p* < 0.05). An increase in handgrip strength values was associated with a decrease in the chances of older adults being classified as SO (*p* < 0.05). The findings suggest that even with SO, sufficiently active older adults did not present a significant reduction in muscle strength in the lower limbs and physical performance.

## Introduction

Sarcopenic obesity (SO) is a condition characterized by the coexistence of sarcopenia and obesity, which can lead to negative consequences on metabolism, the cardiovascular system, muscle strength, and physical performance^1,2^. Such conditions compromise older individuals' functional independence and quality of life^1,2^. SO is often associated with systemic metabolic dysregulation, including insulin resistance and chronic inflammation, which may have direct implications for muscle function^3^. The altered metabolism may compromise the nutrient supply to muscle cells, impacting their ability to generate energy and maintain optimal contractile function^3,4^. In this way, low muscle strength and physical performance exponentially increase the odds of reduced mobility, functional dependence, and frailty in older adults^5,6^. Therefore, therapeutic strategies have been implemented to decrease the deleterious outcomes of SO^7–9^.

The literature reports that a sufficient combination of adequate diet^10^ and moderate-to-vigorous physical activity (≥ 150 min/week of moderate-intensity or ≥ 75 min/week of vigorous, or the combination of both ≥ 150 min/week) may decrease some negative outcomes (mentioned above) of SO in older adults^8^. Numerous studies have investigated the relationship between physical activity and SO, shedding light on their potential benefits in preventing and managing this condition^8,11^. Regular physical activity, including both domestic/household, transportation, occupational, and leisure-time is a significant modifiable factor for the prevention and treatment SO, preventing fat mass gain and increase muscle mass and strength in older adults^11^. Therefore, it is recommended that older adults with SO sustain a sufficiently active lifestyle, with moderate-to-vigorous physical activity levels that exceed resting energy expenditure, and adequate protein intake^10^ to improve their quality of life^8,12^.

Despite the beneficial effects of physical activity, however, adequate levels of physical activity do not exempt older adults from being impacted by the SO phenotype^13^. A recent consensus established by the European Society for Clinical Nutrition and Metabolism (ESPEN) and the European Association for the Study of Obesity (EASO) highlighted that, even with adequate physical activity, SO may still be associated with reduced strength and performance due to increased fat deposits in already-reduced muscle tissue^14^.

Furthermore, failure to recognize that sufficiently active older adults with SO may still have the low muscle strength and physical performance can lead to mistakes in therapeutic intervention strategies^10^. There is currently a lack of research regarding the associations between SO, muscle strength, and physical performance in sufficiently active older adults with SO.

Based on the literature reviewed above, this study aimed to investigate the potential associations between SO and decreased muscle strength and physical performance in sufficiently active older adults. Specifically, this study aimed to investigate the associations of SO based on reduced muscle strength and physical performance in sufficiently active older adults. The hypothesis was that despite experiencing SO, sufficiently active older adults would not display a notable decline in muscle strength, and they would show improved functional test scores.

## Methods

### Study design

This study uses a quantitative correlational-descriptive approach with a cross-sectional design.

### Participants

This is a secondary analysis of a larger project involving older adults^15^. Data from 94 older adults of both sexes, aged 60 to 85 years, who met the inclusion criteria were analyzed. The participants were recruited from the seniors’ physical activity program offered at the School of Physical Education and Sport of Ribeirão Preto, University of São Paulo (EEFERP/USP), for community-dwelling older adults of Ribeirão Preto city and region, and the data were obtained from participants in master projects conducted in our research group^15–17^.

To ensure statistical quality, the sample size was calculated using previously established formulas^18^. For this calculation, the confidence level (Zy = 0.95) was adopted, the variable with the highest variance as a reference (1 repetition maximum knee extension test [standard deviation ± 19.96kg]) and maximum desired error limits (ε ≤ 8.0 kg)^15,18^. A minimal sample of n = 24 for both sexes was identified. The inclusion criteria required participants to be able to walk independently without mobility-limiting diseases or limitations to perform the tests. Additionally, older adults were classified as sufficiently active (≥ 150 min/week of moderate/intense physical activity or ≥ 75 min/week of intense physical activity). Were excluded data from patients diagnosed with cancer, uncontrolled chronic diseases (heart or kidney failure), stroke sequelae, weight loss > 3 kg in the last three months, dementia^15–17^, or those who did not complete all the procedures provided for in the study.

The guidelines and ethical aspects of research with human beings were followed according to the Declaration of Helsinki, the Free and Informed Consent Term signed by each participant was also obtained, being approved by the respective Ethics and Research Committees (CAAE: HC-FMRP-USP: 54345016.6.3001.5440; EEFERP-USP: 54345016.6.0000.5659 e EERP-USP: 23987519.5.0000.5393).

### Measurements

#### Evaluation of cognition and physical activity

To ensure the understanding of the tests by the participants, an assessment of cognition was performed with the Mini-Mental State Examination, a widely used screening tool for cognitive impairment in older adults^19^. The reduced version with a maximum score of 19 was used in this study, and participants who scored 12 or lower were considered to have cognitive impairment^20^. This was important to ensure that the participants were able to understand the instructions and perform the tests accurately.

The International Physical Activity Questionnaire (IPAQ) short-version was used to verify the physical activity level of the participants^21^. The IPAQ questionnaire has been widely used in research and clinical practice to assess physical activity levels in older adults^22^. Participants who accumulated at least 75 min of intense physical activity per week or a combination of moderate and intense physical activity totaling at least 150 min per week, were considered sufficiently active^23^.

#### Anamnesis: sociodemographic characteristics, health risk behaviors, and medication use

An anamnesis who was also conducted to collect sociodemographic, recorded data, health risk behaviors, and medication use information. In the form of an interview, older adults were asked about their education (years of study), marital status (married, widowed, divorced, or single), and monthly income. Health risk behaviors such as alcohol consumption (yes or no), tobacco consumption (yes, no, or ex-smoker), and medication use (type and amount per day) were also assessed. These data are important for the characterization of participants and control of confounding biases in statistical analyses^24^. They are widely used in studies and monitoring of older adults, especially for the identification of risk factors and the development of interventions to promote healthy aging^25^.

#### Nutritional status

The nutritional status was assessed using the Mini Nutritional Assessment Test which evaluates global malnutrition (≤ 17 points), risk of malnutrition (17–23.5 points), and normal nutritional status (24–30 points)^26^.

#### Morphologic dimensions

Body mass (kg) (Filizola®, model Personal, Campo Grande, MS, Brazil) and height (m) (Sanny® Professional—ES2020, Brazil) were measured according to established protocols in the literature^27^. Dual Energy X-ray Absorptiometry (DXA) (Hologic® scanner, model QDR4500W software version 11.2, Bedford, MA) was used to measure appendicular lean soft tissue (ALST) (sum of upper limb lean soft tissue lower right and left sides) and fat mass (FM) from total and regional body scans.

#### Sarcopenic obesity (SO) classification

To confirm the diagnosis of SO, the criteria established by Batsis et al.^28^ were used, which required the presence of alterations in ALST and FM. For men, the criteria were ALST < 19.75kg and FM ≥ 25%, while for women the criteria were ALST < 15.02kg and FM ≥ 35%. Thus, the older adults were allocated into four groups: a) men without SO (♂nSO); b) men with SO (♂SO); c) women without SO (♀nSO); and d) women with SO (♀SO). These criteria have been widely used in other studies with SO older adults^7^ and are currently considered as the valid diagnostic method^1^.

#### Muscle strength measures

##### Estimated one repetition maximum (1RM) of knee extension strength

The knee extension strength of the lower limbs (right and left concomitantly) in a leg extension (Lion Fitness® and LFS model, Brazil) was used to estimate the one repetition maximum in this movement^29^, as previously detailed^15^. First, a warm-up of two sets (8 and 10 repetitions) with lowest loads. After tree-minutes rest, the test was performed at 45% of body mass for women^16^ and 64% for men^16^. For the test, these initial loads were increased or decreased to reach the optimal range of estimative of 1RM (until 10 repetitions) by the Brzycki (1993) Eq.^29^.

##### Peak torque of knee extension strength

The knee extension of the lower limb was tested with an isokinetic dynamometer (Biodex System 4 Pro, USA). The protocol for obtaining the measurement was previously reported^15^. The participants sat on a chair, and the backrest was adjusted to enable the backs of their legs to touch the end of the seat. To prevent additional movements, straps were used to secure the trunk, hip, and the leg being tested (right leg). The chair was positioned to align the lateral epicondyle of the knee with the dynamometer rotation axis. The distal end of the tibia was fixed with Velcro, positioned 0.1 m from the lateral malleolus. The contractile mode of the test was concentric:concentric. The participants performed 10 submaximal repetitions at an angular velocity of 60rad/s for extension and 10 for unilateral flexion of the right knee alternately to familiarize themselves with the protocol, followed by three minutes of rest. Then, five interspersed maximum repetitions were performed for the same movements, registering the torque peak in Nm (Peak torque at 60rad/s knee extension strength).

##### Handgrip strength

Handgrip strength was measured using a Jamar® Manual Dynamometer—model 5030J1. The protocol for obtaining the measurement was previously published^30^. Briefly, the dynamometer was set at the second stage, deemed appropriate for most individuals^31^. Older adults assumed a seated position on an armless chair, maintaining an upright posture with knees and elbows flexed at 90°. The forearm was positioned in neutral, with the wrist in a neutral position and allowed movement up to 30° of extension. The upper limb remained suspended in the air, and the hand was positioned on the dynamometer, held by the evaluator. Three attempts were made with the dominant hand (with a one-minute interval between them), with the highest value in kgf recorded^15^.

#### Physical performance measures

##### Six-minute walking distance

Six-minute walking distance was performed to verify the mobility and aerobic performance, being considered submaximal, safety, and well-tolerated for older adults^32^. The protocol of the test was previously established^33^, and the total distance covered (measurement obtained with the test) was recorded in meters^33^.

##### Timed up and go test

The timed up and go test (TUG) was performed to evaluate functional mobility^31^. The participants were instructed to stand up from a chair, walk three meters at a comfortable speed, turn around, walk back to the chair, and sit down again. The total time to complete the task was recorded, and the test was performed twice, with the best time used for analysis.

##### Usual gait speed

The usual gait speed test was carried out over a course of 4 m monitored by a photocell system (kit FSpeed; FE Sistemas®, Brasil) disposed at the initial/final of the way^15^. The protocol for obtaining the measurement was previously published^34,35^. The procedure was repeated and the average of two attempts was used to record the velocity in meters per second (m/s).

### Statistical analysis

The statistical analysis of the study was conducted in three steps.

Step 1 involved comparing the mean values of muscle strength and physical performance between groups using the 2-tailed independent t-test. The groups compared were ♂nSO versus ♂SO and ♀nSO versus ♀SO. Variables that showed a statistically significant difference in this step were then analyzed further in Step 2.

Step 2 involved verifying the association of SO with muscle strength and physical performance variables using multiple linear regression. The independent variable was SO (0 = nSO; and 1 = SO), and the dependent variables were the variables that showed a statistically significant difference in Step 1. Regression was performed in three blocks, with the first being crude (only SO condition) and the following blocks being adjusted for confounding variables (model 1 [SO condition + sociodemographic characteristics] and model 2 [SO condition + sociodemographic characteristics + nutritional status + number of medications taken + health risk behaviors]).

Step 3 involved verifying the odds ratio (OR) of older adults having SO as the dependent variable using binary logistic regression. The independent variables were the muscle strength and physical performance variables that showed a statistically significant difference in Step 1. Regression was performed in three blocks, with the first being crude (only muscle strength or physical performance variables) and the following blocks being adjusted for confounding variables (model 1 [muscle strength or physical performance variables + sociodemographic characteristics] and model 2 [muscle strength or physical performance variables + sociodemographic characteristics + nutritional status + the number of medications taken + health risk behaviours]).

The normality of data was checked using the Kolmogorov–Smirnov test (n ≥ 30) or Shapiro–Wilk test (n < 30), and outliers were checked using the interquartile range (IQR 1.5). All analyses were performed using the SPSS, v. 20.0 (Inc., Chicago, IL, EUA) with a significance level of α = 5%. Descriptive analysis characterized the sample, including measures of central tendency and a 95% confidence interval. The study also followed the Strengthening the Reporting of Observational Studies in Epidemiology (STROBE) checklist for cross-sectional studies.

### Ethical approval and consent to participate

The guidelines and ethical aspects of research with human beings were followed according to the Declaration of Helsinki, the Free and Informed Consent Term signed by each participant was also obtained. This study was approved by Ethics and Research Committees of Clinical Hospital—Faculty of Medicine of Ribeirão Preto (HC-FMRP-USP) (CAAE: 54,345,016.6.3001.5440), School of Physical Education and Sports of Ribeirão Preto (EEFERP-USP) (CAAE: 54,345,016.6.0000.5659), and Ribeirão Preto College of Nursing (EERP-USP) (CAAE: 23,987,519.5.0000.5393).

## Results

**Figure 1:**
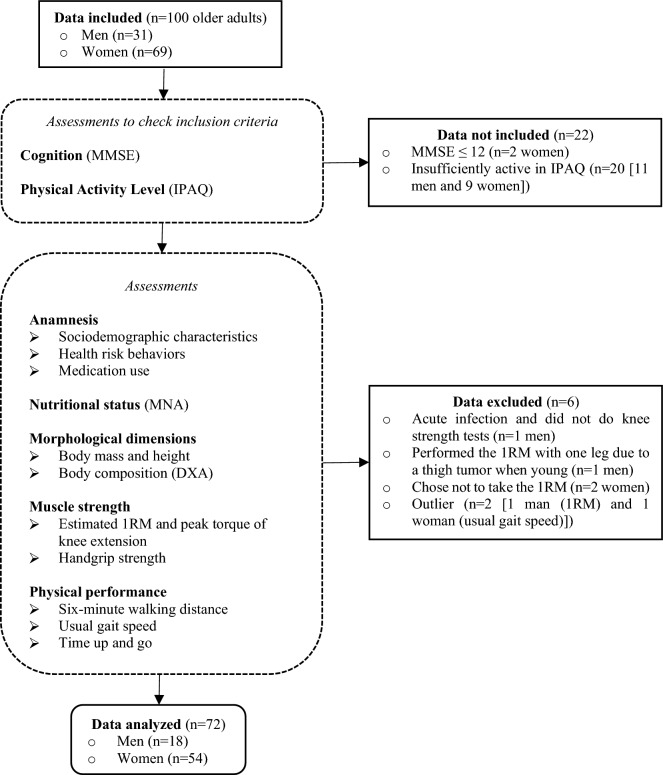
Study phases and data from older adults included, excluded, analyzed, and procedure flow. *Note* MMSE: Mini-Mental State Examination; IPAQ: International Physical Activity Questionnaires; MNA: Mini Nutritional Assessment; DXA: Dual Energy X-ray Absorptiometry; 1RM: one repetition maximum.

After applying the inclusion criteria and detection of discrepant values, 72 older adults were initially included in the study. Of this sample, 44.4% were classified as nSO (n: 32) and 55.6% (n: 40) as SO, based on the criteria previously described. These participants were then included in the final sample for analysis (Fig. 1).

Table 1 provides the descriptive characteristics of the total sample stratified by SO condition. The older adults with SO were found to be significantly older, lighter, and shorter than the older adults without SO. There were no significant differences between the two groups in terms of sex, education level, income, or smoking status. However, a higher proportion of older adults with SO reported being physically inactive and having a lower nutritional status compared to those without SO.

**Table 1:** Descriptive characteristics of older adults with and without sarcopenic obesity (SO) (n = 72).

Variables	♂nSO (n = 11)				♂SO (n = 7)				♀nSO (n = 21)				♀SO (n = 33)			
	Mean	SD	95% CI		Mean	SD	95% CI		Mean	SD	95% CI		Mean	SD	95% CI	
			LL	UL			LL	UL			LL	UL			LL	UL
Sociodemographic characteristics																
Age (years)	68.6	7.5	63.6	73.7	74.3	5.4	69.3	79.3	68.9	6.1	66.2	71.6	70.2	5.6	68.2	72.2
Years of study (years)	12.1	6.7	7.6	16.6	8.6	5.0	4.0	13.2	7.0	4.1	5.2	8.8	9.0	5.0	7.3	10.8
Marital status (%)																
Married				66.7								40.0				
Widower				11.4								29.1				
Divorced				22.2								20.0				
Single				0								10.9				
Monthly income (%)																
< 1 minimum wage				11.1								5.5				
1 minimum wage				61.1								34.5				
2–5 minimum wage				22.2								50.9				
> 5 minimum wage				5.6								9.1				
Morphologic dimensions																
Body mass (kg)	73.9	18.9	61.2	86.6	70.0	7.5	63.1	76.9	70.7	11.9	65.4	76.0	64.0	7.1	61.5	66.5
Height (cm)	170.6	10.3	163.7	177.6	164.0	3.7	160.6	167.4	157.3	7.0	154.2	160.4	155.5	5.5	153.6	157.4
ALST (kg)	22.7	5.3	19.1	26.2	18.7	0.6	18.2	19.3	16.2	2.7	15.0	17.4	13.4	1.3	12.9	13.8
FM (%)	25.7	5.7	21.8	29.5	32.4	6.4	26.5	38.3	39.2	5.0	36.9	41.4	43.0	5.2	41.2	44.9
Nutritional status																
Mini Nutritional Assessment (score)	27.2	3.3	24.9	29.4	26.4	3.9	22.9	30.0	26.3	2.8	25.1	27.5	27.3	2.2	26.5	28.1
Health risk behaviors																
Number of medications taken (quantity)	2.3	1.6	1.2	3.4	2.7	2.2	0.7	4.8	3.8	2.3	2.8	4.9	2.9	2.8	1.9	3.9
Alcohol consumption (%)																
No				44.4								74.5				
Yes				55.6								25.5				
Tobacco consumption (%)																
No				5.6								3.6				
Yes				33.3								78.2				
Ex-smoker				61.1								18.2				
Muscle strength																
1RM of knee extension strength (kg)	67.0	23.2	51.4	82.6	66.2	19.1	48.6	83.9	42.2	19.9	33.4	51.0	40.4	14.2	35.4	45.4
Peak torque 60 rad/s knee extension strength (Nm)	2.3	0.8	1.8	2.9	1.7	0.5	1.2	2.2	1.2	0.6	0.9	1.5	1.3	0.3	1.2	1.4
Handgrip strength (kgf)	39.1	7.7	33.9	44.3	30.4	8.0	23.0	37.9	25.5	4.4	23.6	27.5	23.1	4.4	21.5	24.7
Physical performance																
Six-minute walking distance (m)	523.7	75.6	472.9	574.5	430.6	56.1	378.7	482.4	396.7	117.6	344.5	448.8	425.2	74.4	398.8	451.6
Gait speed (m/s)	1.5	0.4	1.2	1.7	1.3	0.2	1.1	1.4	1.2	0.4	1.0	1.4	1.3	0.3	1.1	1.4
Time up and go (m/s)	8.6	0.8	8.1	9.2	9.6	1.8	7.9	11.2	10.8	3.2	9.4	12.2	10.3	2.7	9.4	11.3

♂**,** men; ♀, women; SO, Sarcopenic obesity; SD, standard deviation; CI, confidence interval; LL, lower limit; UL, upper limit; ALST, appendicular lean soft tissue; FM, fat mass; 1RM, one repetitium maximum.

### First step: between groups comparison

**Figure 2:**
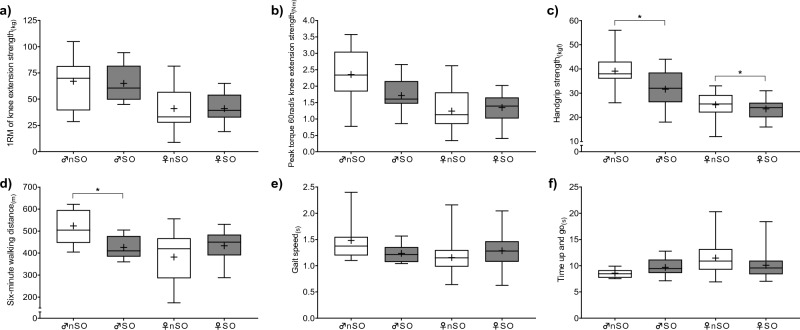
Comparison of muscle strength (**a**, **b**, **c**) and physical performance (**d**, **e**, **f**) between not sarcopenic obese (♂nSO and ♀nSO**)** and sarcopenic obese (♂SO and ♀SO) older adults. *Note:* 1RM = 1 repetition maximum; ♂** = **men; ♀ = women; * = *p* < 0.05.

In the first step of the inferential statistical analysis, a between-groups comparison was conducted. Figure 2 illustrates the comparison of muscle strength and physical performance between older adults without and with SO for both sexes. Among older men, only hand grip strength (t = 2.265) and six-minute walking distance performance (t = 2.993) showed a statistically significant difference between the nSO and SO groups (*p* < 0.05). Similarly, among older women, only hand grip strength (t = 2.037) exhibited a significant difference between the two groups (*p* < 0.05).

### Second step: association parameters of sarcopenic obesity

Table 2 reports the association between SO and muscle strength and physical performance of older adults. Hand grip strength showed a negative association with SO in all models (*p* < 0.001), with R^2^_crude_ = 0.13, SEE_crude_ = 7.2kgf, R^2^_model1_ = 0.53, SEE_model1_ = 5.3kgf, and R^2^_model2_ = 0.54, SEE_model2_ = 5.2kgf. SO was significant (*p* < 0.001) in all models (β_crude_ = − 0.371, β_model1_ = − 0.194, and β_model2_ = − 0.210). The inclusion of sociodemographic information in model 1 increases the coefficient of determination (↑0.40; *p* < 0.001; R^2^_model1_ = 0.53), which was not significant for model 2 (↑0.01; *p* = 0.271). For the six-minute walking distance, only older men showed a significant association (*p* < 0.001) in all models, with R^2^_crude_ = 0.29, SEE_crude_ = 68.9m, R^2^_model1_ = 0.71, SEE_model1_ = 43.7m, and R^2^_model2_ = 0.75, SEE_model2_ = 40.5m. However, SO was significant only in the crude model, losing its significance as the model was adjusted.

**Table 2:** Multivariate linear regression to explain the association of sarcopenic obesity (SO) on hand grip strength and six-minute walking distance (only for men).

Analysis	Model		Adjusted R^2^	SEE	R^2^ change	*p*	Independent variables			
	F	p					Variables	ꞵ		p
Hand grip strength (kgf)										
Crude	11.341	< 0.001	0.13	7.2	–	0.001	SO		− 0.371	0.001
Model 1	14.623	< 0.001	0.53	5.3	0.50	< 0.001			− 0.194	0.033
Model 2	9.475	< 0.001	0.54	5.2	0.01	0.271			− 0.210	0.024
Six-minute walking distance (♂) (m)										
Crude	7.814	0.013	0.29	68.9	–	0.013	SO	− 0.573		0.013
Model 1	9.441	0.001	0.71	43.7	0.42	0.004		− 0.282		0.085
Model 2	6.784	0.006	0.75	40.5	0.04	0.290		− 0.315		0.062

♂ = men; SEE = standard error of estimate; Model 1: adjusted by sociodemographic variables (Age, sex, years of study, marital status, and monthly income); Model 2: adjusted by sociodemographic variables, nutritional status, number of medications taken, and health risk behaviors (alcohol and tobacco consumption).

### Third step: odds to be sarcopenic obese

**Figure 3:**
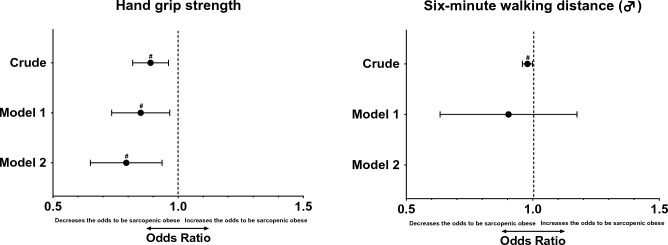
Binary logistic regression model with the association of hand grip strength and six-minute walking distance with sarcopenic obesity (be or not sarcopenic obese). ^#^*p* < 0.05. *Note* OR = odds ratio; CI = confidence interval; LL = lower limit; UL = upper limit; Model 1: adjusted by sociodemographic variables (Age, sex, years of education, marital status, and monthly income); Model 2: adjusted by sociodemographic variables, nutritional status, number of medications taken, and health risk behaviors (alcohol and tobacco consumption). The model 2 in the variable ‘Six-minute walking distance’ was unable to be performed by the software.

Figure 3 presents the OR to be SO. Increase handgrip strength values is associated with a decrease in the OR of older adults being SO, even adjusted by confounding variables (OR: 0.784 [95% CI 0.654–0.940]; *p* < 0.05). Increase the distance traveled in six-minute walking distance was associated with decreasing the OR of older adults being SO for the crude model (OR: 0.979 [95% CI 0.959–0.999]; *p* < 0.05) but does not maintain the statistical significance when the models were adjusted.

## Discussion

This study aimed to investigate the association between reduced muscle strength and physical performance with the risk of SO in sufficiently active older adults. The results of the analysis showed that handgrip strength was different between the sarcopenic obese and the sarcopenic non-obese, for both sexes. Additionally, six-minute walking distance was only different for older men (see Fig. 2). However, there were no differences in other variables of muscle strength, including 1RM of knee extension strength and peak torque 60rad/s knee extension strength, as well as physical performance measures such as six-minute walking distance for older women, gait speed, and time up and go. These findings suggest that while older adults, even those who are sufficiently active, may present reduced upper limb muscle strength, their lower limb muscle strength is not different, and the six-minute walking distance was different only in older men.

Previous studies conducted in other countries have reported associations between SO and muscle strength/physical performance in older adults, but not specifically distinguished by physical activity level. For example, one study of 11,803 older Canadian adults with SO found reduced handgrip strength but no significant difference in gait speed^36^. Another study of older adults with heart failure and SO found reduced physical performance, as measured by short physical performance battery score and 6-min walk distance, and a 2.48 times higher risk of all-cause mortality compared to older adults without sarcopenia/obesity^37^. However, a study of 904 community-dwelling older adults reported no differences between the SO condition and physical performance, as measured by time up and go, sit to stand, one leg stand, walking speed at normal/fastest pace^38^. Instead, an inverse relationship was found between FM and physical performance, suggesting a decrease in physical performance with increased FM^38^. These findings suggest that the relationship between SO and muscle strength/physical performance may vary depending on the population studied and the measures used to assess these variables.

The finding that handgrip strength tends to decrease in individuals with SO while lower extremity muscle strength is often maintained raises questions about the regional distribution of muscle and fat in the body. Recent research suggests that there may indeed be variations in the distribution of fatty infiltration in different muscle groups, contributing to the observed discrepancies in strength across body regions^39–41^. Biomechanically, the increased intramuscular fat in sarcopenic obese individuals may impede the optimal transmission of force along the muscle fibers^42^. Fat is less contractile than muscle tissue, and its presence within the muscle belly may disrupt the coordinated recruitment of motor units, leading to diminished force production^42^. Rahemi et al.^39^ examined the muscle quality and found that obese older adults, particularly in the upper body, exhibited higher levels of intramuscular fat. This suggests that the intramuscular adipose tissue may be more pronounced in the upper limb muscles, potentially contributing to the observed decrease in handgrip strength in older adults with SO.

Handgrip strength is not only a measure of overall muscle strength but is also closely linked to functional independence and quality of life in older adults^43,44^. The reduced handgrip strength in sarcopenic obese individuals may have significant implications for their ability to perform activities of daily living and maintain autonomy^8^. Rolland and Vellas^45^ demonstrated that lower handgrip strength is predictive of functional decline and disability in older adults, highlighting the importance of addressing this parameter in the context of SO^14^. Furthermore, handgrip strength was up to 43.5% lower in Asian community-dwelling older adults with SO than in those who were only obese or only sarcopenic^46,47^. Thus, the present study supports the notion that the coexistence of sarcopenia and obesity during aging is potentially harmful for upper strength parameters^14,48^.

Our study have some strengths The first strength is a large number of muscle strength variables and physical performance measures analyzed. Another strength is the adjustment of regression models with a wide range of confounding factors associated with the odds of being sarcopenic obese, which increases the control of external threats. Furthermore, we compared SO versus nSO groups stratified by sex in an independent t-test. We opted not to use analysis of variance to explore main and interaction effects for sex and SO status due to the potential presence of the Simpson's Paradox. The Simpson's Paradox^49^ occurs when trends appear in different groups of data but disappear or reverse when these groups are combined. In the context of sex and SO status, it is possible that when analyzing the data separately for each sex, significant effects or trends may emerge, but when aggregated, these effects might be obscured or even reversed.

However, the study also has limitations. The cross-sectional design did not allow for the establishment of cause–effect relationships, even more so with the limited sample size. Thus, it is not possible to state whether the absence of differences in lower limb muscle strength and physical performance parameters is due to the older adults being sufficiently active. Additionally, the study only measured upper limb muscle strength through handgrip strength, which does not reflect the overall strength and physical performance of older adults. Despite this, handgrip strength is widely used to identify muscle weakness in older adults, which is a predictive parameter of increased risk of hospital admissions, depression, fractures, and premature mortality^43,44^. Another limitation of our study lies in the dichotomous categorization of alcohol consumption (i.e., 'consume alcohol' and 'do not consume alcohol'), which omits possible dose–response relationships. Moreover, these data are important for the characterization of participants and control of confounding biases in statistical analyses. Lastly, another limitation lies in not including older adults with either sarcopenia or obesity alone. We adopt Batsis et al. (2015) as a reference to the SO diagnostic criteria, which included sarcopenia and obesity alone, and SO. However, our limited sample size restricted the categorization of older adults into only SO and nSO groups. In a way, this may restrict the understanding of whether an additional condition could interfere with the other in terms of muscle strength and physical performance parameters. Therefore, this main point of our article should be considered with caution.

As practical implications of the findings of this study, health professionals responsible for treating SO can explain to older adults that being sufficiently active does not exempt them from suffering reductions in muscle strength (in this study, hand grip strength). In this sense, should seek the advice of licensed exercise professionals to design interventions with resistance exercises to attenuate potential losses in other physical capacities (i.e., lower limb muscle strength and physical performance)^50,51^. Knowing the deleterious effects of SO on handgrip strength (as seen in this study), exercises for upper limbs should be proposed, so that loss of muscle strength over time does not cause impairment in sarcopenic obese older adults^7,52^. This information can help health and exercise professionals in the proper prescription of exercises, even gradually, to increase the risk of SO. It is necessary to consider which physical ability should be improved, as long as the necessary and specific functionality is contemplated^48^. Another implication is possibly including the criterion of upper limb muscle strength (handgrip strength) as a diagnostic criterion for identifying SO. It is discussed about the withdrawal of ALST from DXA from the diagnostic consensus of sarcopenia^53^ since the relationship of this measure with important outcomes (self-reported mobility limitation, falls, fractures, and mortality) for the health of older adults is low^15,44^. Thus, if this tendency is confirmed for the diagnosis of sarcopenia, it may also happen for SO. Thus, handgrip strength could be a surrogate measure for ALST, given its relationship with these relevant outcomes^54^.

For future studies, is suggested longitudinal follow-ups (cohorts) to verify if there is an effect of time on muscle strength and physical performance of older adults with and without SO. In this sense, it would be elucidated whether muscle strength and physical performance could be reduced in older adults with SO, even if they are sufficiently active. In addition, is suggested intervention studies with exercises (i.e., strength, power, and endurance), to verify their impact on body recompositing (fat loss and muscle mass gain) of older adults with SO^52,55^.

## Conclusion

This study determined the associations of sarcopenic obesity from decreased muscle strength and physical performance in sufficiently active older adults. Even with SO, sufficiently active older adults did not present a relevant reduction in muscle strength in the lower limbs and nor in their physical performance. However, older adults with SO had lower upper limb strength, and greater handgrip strength, which was associated with reduced odds of being SO. Therefore, exercise and nutrition strategies should be designed to attenuate losses in upper limb muscle strength, in addition to reversing the diagnosis of SO (increase muscle mass and FM decrease).

### Supplementary Information

Supplementary Information 1.

Supplementary Information 2.

## Data Availability

The datasets generated during and/or analyzed during the current study are available from the corresponding author upon reasonable request.
