# Supplementary material for: Sarcopenic obesity does not impair lower limb strength and physical performance in sufficiently active older adults: a cross-sectional study
Source: Sci Rep. 2024 Feb 6;14:3061. doi: 10.1038/s41598-024-53538-7 (PMC10847493; doi:10.1038/s41598-024-53538-7)
Supplement: Supplementary file 1 — Supplementary Information 1. [file 41598_2024_53538_MOESM1_ESM.docx]

**Supplementary Table.** Multivariate linear regression with multicollinearity diagnosis of each model and confounding variables for handgrip strength and six-minute walking distance (only for men).

| **Analysis** | **Model** | |
| --- | --- | --- |
|  | **Tolerance** | **Variance Inflation Factor** |
| **Hand grip strength (kgf)** | | |
| *Crude* |  |  |
| SO | 1.000 | 1.000 |
| *Model 1* |  |  |
| SO | 0.875 | 1.143 |
| Age | 0.826 | 1.210 |
| Sex | 0.795 | 1.257 |
| Years of study | 0.786 | 1.273 |
| Marital status | 0.938 | 1.066 |
| Monthly income | 0.725 | 1.380 |
| *Model 2* |  |  |
| SO | 0.835 | 1.197 |
| Age | 0.761 | 1.313 |
| Sex | 0.672 | 1.489 |
| Years of study | 0.758 | 1.319 |
| Marital status | 0.912 | 1.096 |
| Monthly income | 0.689 | 1.452 |
| Nutritional status | 0.724 | 1382 |
| Number of medications taken | 0.871 | 1.148 |
| Tobacco consumption | 0.832 | 1.202 |
| Alcohol consumption | 0.712 | 1.404 |
| **Six-minute walking distance (♂) (m)** | | |
| *Crude* |  |  |
| SO | 1.000 | 1.000 |
| *Model 1* |  |  |
| SO | 0.737 | 1.356 |
| Age | 0.549 | 1.821 |
| Years of study | 0.484 | 2.067 |
| Marital status | 0.775 | 1.291 |
| Monthly income | 0.716 | 1.396 |
| *Model 2* |  |  |
| SO | 0.553 | 1.808 |
| Age | 0.518 | 1.932 |
| Years of study | 0.421 | 2.373 |
| Marital status | 0.696 | 1.436 |
| Monthly income | 0.504 | 1.984 |
| Nutritional status | 0.346 | 2.887 |
| Number of medications taken | 0.388 | 2.577 |
| Tobacco consumption | 0.507 | 1.971 |
| Alcohol consumption | 0.488 | 2.050 |
